# Sex Differences in the Association of Depression Symptoms and Cardiovascular Disease in Adults in the United States

**DOI:** 10.1177/08901171241262249

**Published:** 2024-06-12

**Authors:** Bhaskar Thakur, Chance Strenth, Elizabeth Mayfield Arnold, Frank David Schneider

**Affiliations:** 1 12334 Department of Family & Community Medicine, UT Southwestern Medical Center, Dallas, TX, USA; 2 4530 Department of Psychiatry, University of Kentucky, Lexington, KY, USA

**Keywords:** cardiovascular disease, depression, gender, prevalence ratio, The Patient Health Questionnaire-9, health disparities

## Abstract

**Purpose:**

This study explores the relationship between depression and cardiovascular disease (CVD) in the US adult population, focusing on sex differences.

**Design:**

Cross-sectional study.

**Setting:**

National Health and Nutrition Examination Survey data (2013-2018).

**Participants:**

A total of 14 699 community-dwelling adults (≥20 years).

**Measure:**

The Patient Health Questionnaire (PHQ-9) depression screening tool assessed depressive symptoms. CVD events included heart failure, coronary heart disease, angina, heart attack, or stroke.

**Analysis:**

Adjusted prevalence ratios were estimated using a Poisson regression model.

**Results:**

The study finds a positive association between CVD incidents and both mild to moderate depressive symptoms (aPR:1.42, P = .002) and moderately severe to severe depression (aPR:1.72, P = .024). Overall, females exhibit a 47% lower likelihood of CVD incidents compared to males. However, in a subgroup analysis, increased depressive symptoms correlate with higher CVD incidents in females (aPRs range: 2.09 to 3.43, P < .001) compared to males (aPRs range: 1.45 to 1.77, P < .001).

**Conclusion:**

Depression is associated with increased cardiovascular disease (CVD) risk. Females generally have a lower CVD risk than males, but more severe depressive symptoms elevate CVD risk in females. These findings emphasize the significance of considering sex differences. Further research is needed to understand the underlying mechanisms.

## Introduction

Cardiovascular disease (CVD) primarily includes ischemic heart disease (IHD), cardiac arrhythmias, stroke, and congestive heart failure (CHF), and is the leading cause of disability and mortality in the world with 8.9 million deaths attributed to IHD in 2019.^1^ In the United States, CVD accounted for 23% of all deaths in 2019. When direct and indirect costs are included (i.e., loss of productivity), the total cost of CVD in the U.S. by 2035 is estimated to be $1.1 trillion,^2^ and these costs are more commonly shifted to the patients, creating disparities for the uninsured, underinsured, and individuals of low socioeconomic status.^3^ Taken together, CVD has a significant impact on patients’ lives and thus preventive efforts are critical.

Primary care clinicians are uniquely positioned to address the behavioral and physical factors associated with CVD to prevent disease and improve the prognosis but results from interventions to address CVD are mixed. The major modifiable risk factors for CVD are behavioral (e.g., cigarette smoking, diet, sedentary behaviors) that can be altered through interventions.^4^ Other risk factors such as diabetes, hypertension, and high cholesterol can be mitigated through proper medical care.^4^ Primary care is at the forefront of the prevention of CVD, but interventions within primary care targeting multiple behavioral risk factors have produced small to moderate effects that are not clinically significant.^5-7^ A meta-analysis examining the effects of addressing multiple behavioral risk factors in a primary care setting found that these interventions (e.g., motivational interviewing, counseling, and increased PCP visits) did not reduce mortality in CVD patients.^5^

However, emerging research highlights the importance of considering psychological factors, particularly depression, in the prevention and management of CVD. Depression has been shown to be both a risk factor for developing coronary heart disease (CHD) and a comorbid condition that worsens the prognosis.^8^ Several studies have shown that depression is linked with developing various CVD^9^ including myocardial infarctions (MI),^10,11^ CHD,^8,12^ CHF,^11^ stroke,^11-13^ peripheral arterial disease,^13^ and CVD mortality^11,14,15^ in adults. Several studies have highlighted the distinct influence of depression on CVD, with particular emphasis on its impact on the men. In one study, men with higher depressive symptoms had a higher risk of stroke, and stroke mortality at 18-year follow-up.^11,16^ Depression not only leads to an increased risk of developing CVD but also worsens CVD prognosis when a comorbidity exists.^17,18^ For example, men with a previous diagnosis of heart failure had a significantly higher risk of death or cardiovascular hospitalization as their depressive symptoms increased after controlling for other risk factors (e.g., hypertension, body mass index, smoking status, etc.).^19^

Considering the risk that depression poses to the development of CVD and its prognosis, understanding the role of sex is crucial to the prevention of CVD. Despite the risk that CVD poses to women, CVD is still perceived as a disease more likely to occur in men and leads to significant biases such as that women are more protected against CVD risk factors^20-22^ and leads to reduced or lower quality prevention and care.^23,24^ It is important to recognize that societal perceptions often contribute to the misconception that CVD primarily affects men. In 2015, the prevalence of hypertension, CHF, stroke, and atrial fibrillation was higher among females than males.^25^ As a result, medical costs of CVD were also higher among females than males for these four conditions and CVD in total.^25^ Therefore, understanding how behavioral factors such as depression can further exacerbate CVD, especially in women, is critical. Gender or sex differences in depression are well established in the psychological literature, as women more often than men are diagnosed with depression and exhibit more symptoms of depression.^20-22^ Sex differences in the correlation between depression and CVD are associated with variety of biological, psychological, and sociocultural factors (see Penninx^26^ for a review). The reecent proposed mechanisms specific to the relationship between depression and CHD in women include psychosocial, cardiometabolic, behavioral, inflammatory, hormonal, and autonomic factors.^27,28^ The connection between depression and CVD involves a variety of factors, including both behavioral and biological aspects such as increased activity in the sympathetic nervous system and dysfunction in the hypothalamic-pituitary-adrenal axis in women.^27,28^ Recent studies suggest that depression and CVD may be separate clinical conditions affecting different organs (the brain and heart, respectively) but are linked by shared mechanisms, particularly inflammation involving the immune system.^29^ However, data on sex-specific mechanisms in this context are lacking. Prospective studies indicate that inflammatory biomarkers such as CRP and IL-6 predict the occurrence of cardiovascular events in postmenopausal women without pre-existing health conditions.^30^ Additionally, other research suggests that these biomarkers associated with major depressive disorder.^31,32^

A better understanding of the relationship between sex and depression may lead to the development of more targeted preventive interventions to address CVD for women, particularly in primary care settings. Using the National Health and Nutrition Examination Survey (NHANES), we examined the association between depression, sex, and CVD incidents. While prior research led us to expect a higher prevalence of CVD incidents in men, our focus was on exploring the association between depressive symptoms and CVD incidents. Specifically, we hypothesized a positive association, anticipating that individuals with higher levels of depressive symptoms, particularly women, would demonstrate a greater occurrence of CVD incidents compared to men. Our study is cross-sectional, meaning we are not suggesting a cause-and-effect relationship.

## Methods

### Design

Data were used from the 6 years of NHANES data (2013-2014, 2015-2016, 2017-2018). NHANES is a publicly available open survey overseen by the National Center for Health Statistics. NHANES is designed to represent the civilian non-institutionalized population and contains data on demographic, socioeconomic, diet, and health-related problems. (https://www.cdc.gov/nchs/nhanes/index.htm). This study is not considered human research, as determined by the UT Southwestern Medical Center Human Research Protection Program and does not require IRB approval or oversight. Additionally, the NCHS Research Ethics Review Board approved the NHANES protocol, and each participant provided written informed consent prior to data collection.

### Sample

For this study, we utilized 6 years of NHANES data (2013-2014, 2015-2016, 2017-2018) from 14 699 U.S. adults from 29 400 participants selected through a complex probability sampling design.

**Figure 1.**
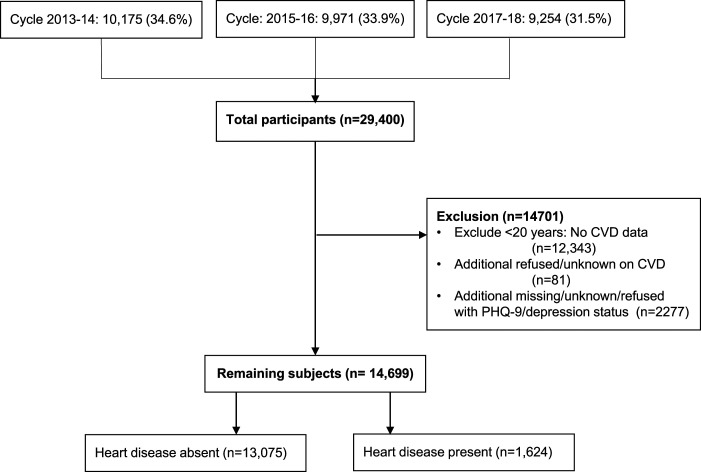
Flow chart outlining the participants’ selection process for the study.

We excluded individuals below the age of 20 years (mostly children and adolescents: n = 12 343), as well as those with refused or unknown status on CVD (n = 81) and those with missing, refused, or unknown status on depressive symptoms (n = 2277). The exclusion process was conducted in a stepwise manner. Initially, the individuals below the age of 20 years were excluded due to the limited CVD data for individuals aged 20 years and older in the NHANES medical conditions’ questionnaire. Among the remaining participants, 81 either refused or reported unknown status on CVD, and subsequently, we excluded 2277 samples due to missing, refused, or unknown status on depressive symptoms. The analysis used the remaining data from 14 699 adults’ data. Figure 1 depicts a flow chart outlining the selection process for inclusion of participants for the study.

## Measures

### Depression Status

The NHANES examined depression using the Patient Health Questionnaire–9 (PHQ-9),^33,34^ a reliable and validated depression screening tool that assesses how often individuals experience symptoms of depression within the past 14 days using a 4-point Likert scale with scores ranging from 0 to 27. Scores are categorized as “No Depression” (score of 0), “Minimal Depression” (1-4), “Mild Depression” (5-9), “Moderate Depression” (10-14), “Moderately Severe Depression” (15-19), and “Severe Depression” (20-27).^35,36^ To facilitate a more meaningful analysis, we merged categories to create three categories, combining ‘No Depression’ with ‘Minimal Depression’, ‘Mild Depression’ with ‘Moderate Depression’, and ‘Moderately Severe Depression’ with ‘Severe Depression’. This modification was made due to small frequencies in the “Moderately Severe” and “Severe Depression” categories.

### CVD Incidents

In our analysis, a CVD incident was defined as the occurrence of any of the following conditions: CHD, MI, Angina Pectoris, CHF, or Stroke, as defined in the NHANES surveys.

Information on sex and other covariates is made available in Supplemental Materials.

### Analysis

To adhere to the NHANES Analytic and Reporting Guidelines^37^ specified by the CDC, we integrated the combined sample weights for the NHANES cycle 2013-2014, 2015-2016, and 2017-2018 in our statistical analyses.

To summarize the sociodemographic characteristics, lifestyle factors, and medical conditions, the weighted mean and standard deviations were used to represent continuous variables including age and BMI. For the categorical variables including sex, ethnicity, marital status, number of people in the household, education, smoking status, income, metabolic syndrome, obesity, depression, and CVD status, weighted frequencies, and proportions (%) were calculated. To compare these parameters between individuals with and without CVD and determine if there were significant differences, we used the adjusted Wald test for quantitative parameters such as age and BMI. For categorical parameters, we employed the second-order Rao-Scott Pearson Chi-square test statistic (design-based F test).

We conducted survey-based generalized linear models with the Poisson family and log link function (standard errors were estimated by using the linearized variance estimator^38^) to examine the potential links between sex, depression, and CVD incidents and reported the prevalence ratios (PRs) and 95% confidence intervals (CIs). The PR is a robust measure of effect size in cross-sectional data and asserts only an association, not a causal relationship. A *P-*value of less than .05 was considered statistically significant for all statistical comparisons. All the analysis was performed using Stata 18.0 MP—Parallel Edition.^39^ Method on the analysis of missing data and detailed statistical analysis is provided in the Supplemental Material.

## Results

The study sample consisted of 14 699 adults. The mean age was 47.9 years (SD = 17.1) and the average BMI was 29.5 (SD = 7.1). Additionally, about half (51.5%) of the participants identified as female (Table 1). In terms of race and ethnicity, the majority were NH white (65.1%), followed by Hispanic (15%), NH black (11.1%), NH Asian (5.1%), and NH multiracial (3.7%). Other demographic characteristics, including marital status, education, smoking status, income, and household size are shown in Table 1. A total of 47.1% had metabolic syndrome, while 40.1% were classified as obese. CVD was present in 8.9% of participants, and depression was reported as mild/moderate in 21.1% and moderately severe/severe in 2.9%.

**Table 1.** Participant’s Sociodemographic, Clinical Characteristics, Depression Status, and Their Relationship With Cardiovascular Incidents.

Demographic and clinical characteristics	Overall (N = 14 699)	Without CVD incident (n:13 075; %:91.1)	With CVD incident (n:1624; %:8.9)	*P*-value
Age, mean (SD)	47.9 (17.1)	46.3 (16.4)	64.9 (14.0)	<.001
BMI, mean (SD)	29.5 (7.1)	29.4 (7.0)	31.1 (8.4)	<.001
Female, frequency (%)				
No	7139 (48.5)	6212 (90.0)	927 (10.0)	.001
Yes	7560 (51.5)	6863 (92.1)	697 (7.9)	
Ethnicity, frequency (%)				
Mexican American/other Hispanic	3696 (15.0)	3404 (94.9)	292 (5.1)	<.001
Non-Hispanic white	5569 (65.1)	4758 (90.1)	811 (9.9)	
Non-Hispanic black	3173 (11.1)	2801 (90.7)	372 (9.3)	
Non-Hispanic Asian	1680 (5.1)	1599 (95.9)	81 (4.1)	
Non-Hispanic multiracial	581 (3.7)	513 (87.3)	68 (12.7)	
Marital status, frequency (%)				
Never married	2710 (18.3)	2577 (96.2)	133 (3.8)	<.001
Married	7477 (54.7)	6686 (91.3)	791 (8.7)	
Living with partner	1280 (8.7)	1202 (95.2)	78 (4.8)	
Widow/divorced/separated	3224 (18.3)	2603 (83.4)	621 (16.6)	
Unknown/missing	8 (.0)	7 (81.7)	1 (18.3)	
Education, frequency (%)				
Lower than high school	3043 (12.9)	2622 (87.8)	421 (12.2)	<.001
High school graduate or GED	3365 (23.4)	2929 (89.4)	436 (10.6)	
Some college or AA degree	4615 (32.3)	4114 (91.0)	501 (9.0)	
College graduate or above	3668 (31.4)	3403 (93.8)	265 (6.2)	
Unknown/missing	8 (.0)	7 (94.7)	1 (5.3)	
Smoked at least 100 cigarettes in life, frequency (%)				
No	8359 (56.5)	7733 (93.9)	626 (6.1)	<.001
Yes	6332 (43.4)	5334 (87.4)	998 (12.6)	
Unknown/missing	8 (.1)	8 (100.0)	0 (.0)	
Income, frequency (%)				
lower than 45k	7171 (38.4)	6135 (87.4)	1036 (12.6)	<.001
45-100k	3867 (30.2)	3537 (92.4)	330 (7.6)	
>100k	2416 (24.3)	2284 (95.5)	132 (4.5)	
Unknown/missing	1245 (7.1)	1119 (90.5)	126 (9.5)	
Metabolic syndrome, frequency (%)				
No	6843 (50.1)	6423 (95.1)	420 (4.9)	<.001
Yes	7372 (47.1)	6214 (86.6)	1158 (13.4)	
Unknown/missing	484 (2.8)	438 (93.4)	46 (6.6)	
Obesity, frequency (%)				
Underweight	210 (1.4)	190 (92.8)	20 (7.2)	<.001
Normal weight	3766 (26.0)	3459 (94.0)	307 (6.1)	
Overweight	4680 (31.7)	4175 (91.4)	505 (8.6)	
Obese	5899 (40.1)	5151 (89.2)	748 (10.8)	
Unknown/missing	144 (.8)	100 (73.8)	44 (26.2)	
Number of people in the household, frequency (%)				
1	2073 (13.1)	1657 (83.9)	416 (16.1)	<.001
2	4362 (33.8)	3717 (88.0)	645 (12.0)	
3	2670 (18.7)	2450 (93.8)	220 (6.3)	
4	2327 (16.3)	2164 (94.5)	163 (5.5)	
5	1647 (10.2)	1559 (96.4)	88 (3.6)	
6	851 (4.5)	804 (95.9)	47 (4.1)	
7 & more	769 (3.4)	724 (95.5)	45 (4.5)	
Depression, frequency (%)				
No/minimal depression	10 960 (76.0)	9984 (92.9)	976 (7.1)	<.001
Mild/moderate depression	3245 (21.1)	2713 (86.3)	532 (13.7)	
Moderately-severe/severe depression	494 (2.9)	378 (79.1)	116 (20.9)	

A comparative analysis, detailed in Table 1, was performed to examine the differences between individuals diagnosed with CVD incidents and those without CVD incidents. Individuals with CVD incidents had a significantly higher mean age (64.9 ± 14.0) compared to those without CVD incidents (46.3 ± 16.4). Similarly, the mean BMI was slightly higher among individuals with CVD incidents (31.1 ± 8.4) compared to those without CVD incidents (29.4 ± 7.0). Among the study participants, a significantly lower percentage of females as compared to males had CVD incidents (7.86% vs 10%). Regarding race/ethnicity, individuals of NH multiracial background had a higher percentage of CVD incidents (12.7%) followed by NH white (9.89%), NH black (9.31%), Hispanic (5.11%), and NH Asian (4.11%). Significant differences were also observed in other social indicators such as marital status, education, smoking status, income, presence of metabolic syndrome, obesity, and number of people in the household. Individuals with CVD incidents were more likely to have a marital status of divorced/widowed/separated, had lower education levels, a history of smoking, lower income, and the presence of metabolic syndrome and obesity (Table 1). The prevalence of CVD incidents was 7.13% among individuals without depression or with minimal symptoms, 13.7% among those with mild/moderate depression, and 21% among individuals with moderately severe/severe depression (*P* < .001).

**Figure 2.**
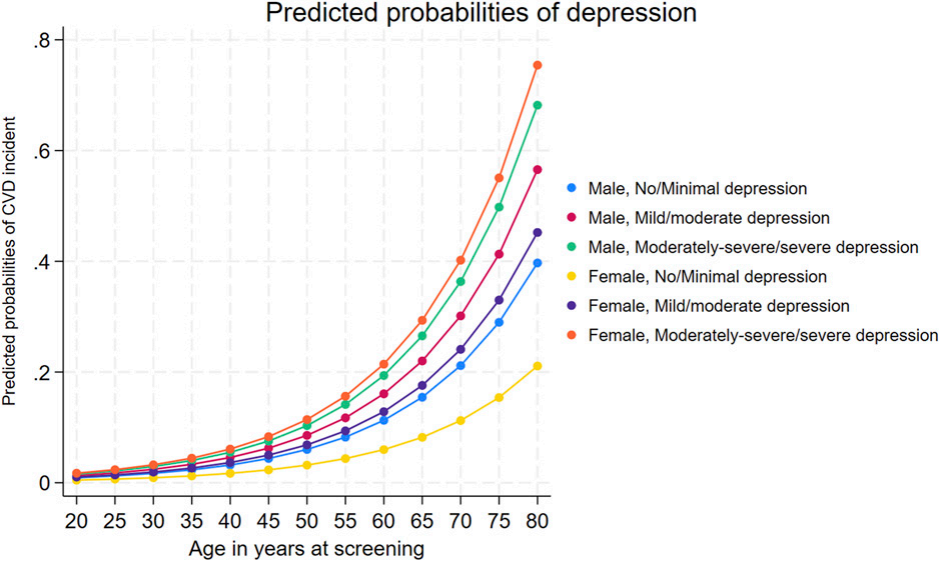
Age-specific probabilities of cardiovascular disease by depression class: A sex comparison.

The unadjusted and adjusted prevalence ratios (PRs) are presented in sTable 1 under supplementary material which show a significant association between depression, sex, and CVD incidents. In the unadjusted analysis, a higher level of depression was positively associated with an increased prevalence of CVD incident (uPR: 1.61; 95% CI: 1.29, 2.01; *P* < .001 for mild to moderate, and uPR: 2.01; 95% CI: 1.33, 3.03; *P* = .001 for severe groups, respectively). Additionally, females had a 41% lower likelihood of experiencing a CVD incident compared to males. However, upon examining the interaction effect within the same model, we observed significantly higher uPRs for CVD incident (*P* < .05) among females in the mild to moderate (uPR: 1.58; 95% CI: 1.16, 2.15; *P* = .005) and moderately severe to severe (uPR: 2.16; 95% CI: 1.36, 3.44; *P* = .002) depression categories compared to males with no or minimal depressive symptoms. These associations persisted in the adjusted analysis, which controlled for multiple covariates including age, ethnicity, marital status, education, family income, household size, smoking status, obesity, and metabolic syndrome (sTable 1). While understanding the interaction effects in our study can be a bit complex, the fact that these interactions are statistically significant means that both depression and sex play a combined role in influencing the likelihood of CVD incidents. We have taken a practical approach by showing the chances of experiencing CVD incidents for different combinations of depression levels and sex in sTable 2 under supplementary material. The unadjusted and adjusted estimated probabilities clearly showed that the likelihood of depression incidents in males is high compared to females in the group with null to moderate levels of depression. However, this likelihood was found to be increased in females compared with males in the group experiencing moderately severe or severe depression. Figure 2 displays the age-specific adjusted probabilities of CVD incidents, categorized by sex and various depression levels. It clearly illustrates that the probability of CVD incidents increases with age and remains consistently higher in females experiencing moderately severe or severe depression.

In subpopulation-unadjusted and adjusted analyses, we observed that depressive symptoms among males and females both were positively and significantly associated with a higher likelihood of CVD incidents (Table 2). However depressive symptoms in the females were associated with a double effect size of higher CVD incident (aPR:2.09; 95% CI: 1.70, 2.58; *P* < .001 for mild to moderate, and PR:3.43; 95% CI: 2.71, 4.35; *P* < .001 for severe group respectively) as compared to males (PR:1.45; 95% CI: 1.19, 1.77; *P* < .001 for mild to moderate, and PR:1.77; 95% CI: 1.23, 2.54; *P* = .003 for severe group respectively).

**Table 2.** Subgroup Analysis of the Sex-Based Association Between Depression and Cardiovascular Disease Incidents.

Subpopulation	Parameters of interest	uPR (95%CI)	*P*-value	aPR^a^ (95%CI)	*P*-value
Male	Depression level				
	Mild/moderate vs no/minimal	1.61 (1.29, 2.01)	<.001	1.45 (1.19, 1.77)	<.001
	Moderately severe/severe vs no/minimal	2.01 (1.33, 3.02)	.001	1.77 (1.23, 2.54)	.003
Female	Depression level				
	Mild/moderate vs no/minimal	2.54 (2.03, 3.17)	<.001	2.09 (1.70, 2.58)	<.001
	Moderately severe/severe vs no/minimal	4.35 (3.37, 5.61)	<.001	3.43 (2.71, 4.35)	<.001

Abbreviation: uPR, unadjusted prevalence ratio; CI, confidence interval; aPR, adjusted prevalence ratio.

^a^Accounts for controlling multiple covariates, including age, ethnicity, marital status, education, family income, household size, smoking status, obesity, and metabolic syndrome.

## Discussion

The present study provide insights into the association between depression, sex, and CVD incidents, as well as the interaction between sex and depression associated with CVD incidents in the United States adult population. Comparative analyses revealed significant differences between individuals with and without CVD. The group with CVD was older, had a higher BMI, and included a lower proportion of females compared to those without CVD incidents. Race/ethnicity also appeared to play a role, with NH multiracial individuals having the highest prevalence of CVD. These findings support previous research, emphasizing the significance of these factors on CVD incidents.^40-42^ Study findings reveal higher rates of CVD incidents among individuals with depressive symptoms, particularly among females even after controlling for the potential covariates including demographic characteristics (age, race, ethnicity, household size), social determinants of health (marital status, educational attainment, and annual household income), lifestyle factors (smoking, alcohol intake, and obesity), and metabolic syndrome. This finding highlights a notable sex differences, suggesting that women are more volnurable to CVD across the varying levels of depression severity then men. The meaning of these findings along with their implications are discussed in the following paragraphs along with the limitations of our study and finally our conclusion.

In particular, our findings provide important information about the potential association of depression with CVD which aligns with previous research documenting that depression is a significant risk factor for developing CVD.^9,43-45^ Individuals with a history of heart failure and increasing depressive symptoms are at a higher risk of death or cardiovascular hospitalization.^43^ In this study, about one-fourth of the sample had some level of depressive symptoms, but only a small percentage (less than 3% had more severe symptoms). Nonetheless, this finding is concerning, particularly given that left untreated, symptoms may increase over time. Depression worsens the prognosis of CVD, as individuals with a history of heart failure and increasing depressive symptoms are at a higher risk of death or cardiovascular hospitalization.^9,46^

We observed sex differences in the association between depression and CVD, with a significantly stronger association among females compared to males. In our study, we observed from our interaction analysis that sex differences in the association between depression symptom and CVD incidents, with a significantly stronger association among females compared to male. In Particular, the age-specific adjusted probabilities of CVD incidents not only increased with age in various depression categories but also remained consistently higher in females experiencing moderately severe or severe depression. Moreover, the observed larger effect size in the female subsample supported by the distinct and almost non-overlapping 95% confidence intervals (in both unadjusted and adjusted prevalence ratios) from the male subsample suggests a significantly stronger association between depressive symptoms and CVD events among females compared to males. In response to concerns about our analyses showing interaction by sex, the existing literature presents diverse perspectives on the relationship between depression and CVD in different sex. Our investigation aligns with other findings in the literature. Notably, a recent narrative review highlighted a robust connection between CHD and the prevalence of depression in women compared to men.^27^ Furthermore, an additional review emphasized that, in females, the co-occurrence of depression and CVD is associated with a more severe degree of illness and prognosis compared to isolated diagnoses.^47^ Data from the literature consistently point towards a heightened susceptibility of women to CVD incidents when experiencing depression. This is underscored by a doubled incidence of CVD-related deaths, along with increased prevalence of angina, heart failure, and stroke in females.^28^ A study conducted by Ferketich et al^48^ highlighted that depression correlates with a heightened risk of CHD incidence, demonstrating equal impact in both men and women. Moreover, the presence of major depression among patients with recent acute MI or unstable angina significantly amplifies the risk of cardiac death, with the hazard more than doubling for both sexes.^49^ A review emphasized that although stress impacts cardiovascular health metrics in both men and women, its effect on measures of glucose regulation and dyslipidemia, as well as overall CVD risk, may be more pronounced in women.^50^ A recent study from Japan suggests a significant association between depression and subsequent CVD events in both sexes, with a more pronounced association observed in women.^51^ Another Korean study emphasized that the presence of depression was not associated with CVD risk regardless of sex after controlling for confounding factors.^52^ However, this is important to highlight the evidence of a stronger association between depression and stroke in men observed in a study conducted by Hamano and colleagues in Sweden using electronic health records.^53^ A research conducted in India indicated that men with multiple CVDs faced a higher risk of major depressive disorder compared to women with CVDs.^54^ These findings emphasize the complex relationship between depression and CVD incidence with respect to sex. The exploration of sex differences in the association between depression and CVD remains vital due to the heterogeneous nature of the findings. While some studies indicate a heightened association between depression and CVD in women, others suggest an equal association, and some even propose a heightened association in men. Our findings highlight the importance of considering sex differences in depression when assessing CVD risk in clinical settings. This is also important to consider moving forward when developing clinical interventions for CVD since historically men or males have been favored over women as intervention test subjects or participants across the field of medicine with the National Institute of Health (NIH) enacting the sex as a biological variable policy in order for researchers to consider women within clinical interventions.^55^

Using the NHANES dataset, Shen et al^56^ employed a traditional weighted logistic regression approach, and this study dropped the missing cases for various covariates and focused solely on CHD and stroke in analyses, potentially contributing to the non-significant observation of interaction between sex and depression. Reporting prevalence ratios (PRs) can offer a robust examination of potential links between sex, depression, and CVD incidents in a cross-sectional dataset. In a separate analysis using the NHANES linked with mortality data, Zhang et al^57^ conducted a time-to-event analysis, considering death from all causes, CVD, and IHD, and reported no significant interaction effect between sex and depression on mortality. However, our analysis reveals that sex significantly influences the relationship between depression and the occurrence of cardiovascular incidents, offering unique insights distinct from the mortality-centric approach. A recent meta-analysis highlighted that both women and men with depression exhibit similar effects on incident IHD.^58^ In contrast, our study reveals a significant likelihood of CVD incidents among females with moderately severe or severe depression, a trend that remains consistent across various age groups. We also observed that the effect of association between depression and CVD in the subpopulation of females is higher than in males.

The aforementioned studies have demonstrated linkages between depression and CVD, including its poor prognosis and exacerbation. have addressed potential confounding factors, with adjustments differing across the literature.^28,43,44,46,53^ In Lee et al^43^ study, adjustments were made for additional parameters such as family poverty income ratio (PIR) and physical activity measured in MET-min/week, which were not directly controlled in our study. However, this study did not control other important covariates including race, ethnicity, and obesity. Instead of the family PIR, we utilized household income and household size, which could serve as similar adjustments. It’s important to note that detailed measures of similar physical activity were largely unavailable (>50% missing) in the NHANES cycles used for our analysis. In another study carried out by Rajan et al,^44^ they adjusted for some additional parameters such as urban/rural residence, self-reported disabilities, social isolation index, unhealthy diet, adverse life experiences, and antidepressant use, which were not directly controlled in our study due to unavailability. However, they did not control other potential covariates including marital status, household income, household size, and metabolic syndrome. Moreover.

The association between depression and CVD can be attributed to multiple behavioral and physiological pathways. Depression influences CVD risk behaviors such as diet, sedentary behavior, and smoking. Physiologically, depression dysregulates the hypothalamic-pituitary-adrenal (HPA) axis,^59^ activates the sympathetic nervous system,^60^ and increases inflammation,^61^ all of which can exacerbate CVD.^62-64^ Implementing preventive depression strategies in primary care can enhance mental well-being which can be beneficial to minimize the impact of the above pathways and potentially mitigate the associated inflammation and dysregulation.

## Limitations

There are several limitations to consider when interpreting the results of this study. First, the cross-sectional nature of the NHANES data limits causal inferences. The relationship could be bidirectional; however, we have used the prevalence ratio to report the association between depression and CVD events. Also, our major focus is to highlight the sex differences in this association. Longitudinal studies are needed to establish temporal relationships between depression and CVD outcomes. Second, although efforts were made to control potential confounding factors including age, race, ethnicity, household size, marital status, educational attainment, annual household income, smoking, alcohol intake, obesity, and metabolic syndrome, and most of these factors align with the existing studies, it’s important to acknowledge that covariates not included in our study might influence the relationship between depression, sex, and CVD incidents. Controlling additional covariates including but not limited such as medical history and comorbidities, functional/disability status, country of origin, family history, health insurance, physical activity, urban/rural residence, social isolation index, unhealthy diet, adverse life experiences, and antidepressant use are worth to explore and control for potential confounders in the relationship of depression symptom and CVD outcomes. Third, the generalizability of the findings may be limited to the study sample, of adults aged 20 and above in the United States. Diverse populations with varying characteristics and cultures may have different associations between depression and CVD. Finally, the reliance on self-reported CVD status and depression symptoms, and potential underdiagnosis or misclassification could have introduced measurement errors. Future research could benefit from utilizing medical records or structured clinical interviews to assess the relationship between CVD incidents and depression. Despite these limitations, this study offers valuable insights into the connection between depression, sex, and CVD incidents.

One of the strengths is that its findings can be applied broadly because NHANES selection procedures are well-planned, ensuring the sample reflects the study population accurately. This allows for valuable insights into depression, sex, and CVD, opening the door for further exploration in longitudinal studies.

Our study highlights the complex relationship between depression, sex, and CVD incidents among adults in the United States. We identified significant sex disparities, with females exhibiting a heightened likelihood to CVD across varying levels of depression severity symptoms compared to males. These findings highlight the need for better screening and interventions for depression and CVD, especially among more vulnerable groups, such as females with depressive symptoms. It is also important to modify approaches based on sex-specific factors in both clinical and public health settings to meet the distinct needs of male and female patients. Moreover, focusing on depression as a factor that can be changed through preventive strategies could help reduce its impact on CVD risk, stressing the importance of early intervention in primary care settings.

**So What?**

**What is Already Known on This Topic?**

CVD is a major global health concern exacerbated by modifiable risk factors like smoking and hypertension. Previous research has established depression as a significant risk factor for CVD development and prognosis, with recognized sex differences.

**What Does This Article Add?**

This study examines how depression and sex affect CVD using national data. It emphasizes women’s higher CVD risk with depression, urging attention to sex differences in mental health for heart health efforts.

**What are the Implications for Health Promotion Practice or Research?**

Health care providers should integrate mental health assessments and interventions into CVD prevention strategies, especially targeting high-risk populations like women with depression. Future research should focus on longitudinal studies to better understand the temporal relationship between depression and CVD outcomes, informing the development of more effective integrated care approaches.

## Supplemental Material

Supplemental Material - Sex Differences in the Association of Depression Symptoms and Cardiovascular Disease in Adults in the United States Supplemental Material for Sex Differences in the Association of Depression Symptoms and Cardiovascular Disease in Adults in the United States by Bhaskar Thakur, Chance Strenth, Elizabeth Mayfield Arnold, and F. David Schneider in American Journal of Health Promotion.
