# Supplementary material for: Sex Differences in the Association of Depression Symptoms and Cardiovascular Disease in Adults in the United States
Source: Am J Health Promot. 2024 Jun 12;38(8):1199–209. doi: 10.1177/08901171241262249 (PMC11528955; doi:10.1177/08901171241262249)
Supplement: Supplemental Material - Sex Differences in the Association of Depression Symptoms and Cardiovascular Disease in Adults in the United States [file sj-pdf-1-ahp-10.1177_08901171241262249.pdf]

Sex and Covariates

Before conducting the analysis, covariates were predetermined based on prior evidence of sociodemographic and clinical considerations. These covariates included demographic characteristics of the participants (age, sex at birth, race, ethnicity, household size), social determinants of health (marital status, educational attainment, and annual household income), lifestyle factors (smoking, alcohol intake, and obesity), and metabolic syndrome. Age was considered as a continuous variable. Sex was categorized as male and female; and self-reported race and ethnicity were categorized according to NHANES protocol, including, Mexican American/other Hispanic, non-Hispanic (NH) White, NH Black, non-NH Asian, NH multiracial. Marital status was divided into four groups (never married, married, living with partner, widow/divorced/separated); education was classified into four groups (less than high school, high school graduate, some college, and college graduate or higher); and annual household income was categorized as <$45,000, $45,000 to $100,000, and ≥$100,000. Smoking was defined as smoking at least 100 cigarettes in one's lifetime, alcohol intake was defined as having at least 12 alcohol drinks in 1 year. Based on BMI measured in kg/m2, obesity status was defined as underweight (<18.5), normal (≥18.5 & < 25.0), overweight ((≥25.0 & < 30.0), and obese (> 30.0). Metabolic syndrome was defined as the presence of a minimum of any three of the following: increased blood pressure, high blood sugar, excess body fat around the waist, and abnormal cholesterol or elevated triglyceride levels.

Analysis of Missing Data

Missing or unreported information, as well as unknown values in other covariates, were consolidated and treated as a distinct category. Subsequently, we analyzed the data, considering these entries as not missing primarily to control confounding variables. We observed that the information for this distinct category is less than 5% for any covariate, except for income, where the missing rate is approximately 7% (Table 1). It is important to note that interpretations for results arising from these specific data points in the model were not provided. Due to the cross-sectional nature of the data, we lack sufficient information to impute these missing parameters. Nevertheless, the robustness of our findings is supported by the large sample size and the application of rigorous statistical methods.

Detailed Statistical Analysis

It is recommended to choose either the Cox or Poisson regression model with a robust variance estimator to estimate the prevalence ratio. However, the survey-based model by default computes standard errors by using the linearized variance estimator which is based on a first-order Taylor series linear approximation[28] which is referred to as equivalent to a robust variance estimator in a non-survey context. We incorporated an interaction term into our analysis to systematically examine potential variations in the association between depression and CVD across sex. This interaction term encompasses both the main effects of the variables and their interaction, allowing us to explore how the relationship between depression and CVD may differ between male and female participants. To account for potential confounding factors, we used a similar generalized linear model to adjust for various covariates in the models.

After estimating PRs through the mentioned approach, we further calculated marginal probabilities to enhance the interpretability of our results. These probabilities were derived from the predicted counts generated by the Poisson regression model, providing the likelihood of CVD incidents for various combinations of depression levels and sex. We also demonstrated sex-wise age-specific probabilities of CVD incidents by depression class. Furthermore, we conducted survey-based generalized linear models with the Poisson family and log link function to find out the association between depression status and CVD incidents in a subpopulation of male and female sex.

sTable 1. Interaction effects of sex and depression symptoms on cardiovascular disease status.

Parameters of interest	uPR (95% CI)	P value	aPR* (95% CI)	*p*-value
**Depression level**				
Mild/moderate ***vs.*** no/minimal	1.61 (1.29, 2.01)	< 0.001	1.42 (1.17, 1.73)	0.001
Moderately severe/severe ***vs.*** no/minimal	2.01 (1.33, 3.03)	0.001	1.72 (1.19, 2.48)	0.005
**Sex**				
Female ***vs.*** male	0.59 (0.49, 0.70)	< 0.001	0.53 (0.44, 0.64)	< 0.001
**Interaction between sex & depression**				
Female with Mild/moderate levels of depression ***vs.*** normal male	1.58 (1.16, 2.15)	0.005	1.50 (1.15, 1.98)	0.004
Female with moderately severe/severe depression level ***vs.*** normal male	2.16 (1.36, 3.44)	0.002	2.08 (1.33, 3.25)	0.002

Abbreviation: uPR, unadjusted prevalence ratio; CI, confidence interval; aPR, adjusted prevalence ratio.
*: accounts for controlling multiple covariates, including age, ethnicity, marital status, education, family income, household size, smoking status, obesity, and metabolic syndrome.

sTable 2. Predicted unadjusted and adjusted probabilities of cardiovascular disease incidents.

		Unadjusted			Adjusted*		
Sex	Depression level	P (CVD)	95 % CI		P (CVD)	95 % CI	
Male	No or Minimal	0.09	0.08	0.10	0.10	0.09	0.11
Male	Mild or moderate	0.14	0.12	0.17	0.14	0.12	0.16
Male	Moderately severe or severe	0.18	0.10	0.25	0.17	0.11	0.22
Female	No or Minimal	0.05	0.04	0.06	0.05	0.04	0.06
Female	Mild or moderate	0.13	0.11	0.16	0.11	0.09	0.13
Female	Moderately severe or severe	0.23	0.17	0.29	0.19	0.14	0.23

Abbreviation: P (CVD), probability of cardiovascular disease incident; CI, confidence interval.
*: accounts for controlling multiple covariates, including age, ethnicity, marital status, education, family income, household size, smoking status, obesity, and metabolic syndrome.
